# The MemClin project: a prospective multi memory clinics study targeting early stages of cognitive impairment

**DOI:** 10.1186/s12877-020-1478-3

**Published:** 2020-03-06

**Authors:** Urban Ekman, Daniel Ferreira, J-Sebastian Muehlboeck, John Wallert, Anna Rennie, Maria Eriksdotter, Lars-Olof Wahlund, Eric Westman

**Affiliations:** 1 Department of Neurobiology, Care Sciences, and Society, Division of Clinical Geriatrics, Karolinska Institutet, Stockholm, Sweden; 2 Theme Aging, Karolinska University Hospital, Huddinge, Sweden; 3 Department of Women’s and Children’s Health, Uppsala University, Uppsala, Sweden; 4 Department of Neuroimaging, Centre for Neuroimaging Sciences, Institute of Psychiatry, Psychology and Neuroscience: King’s College London, London, UK

**Keywords:** Cognitive impairment, Dementia, Diagnostics, Prognostics, Neuropsychology, Biological markers

## Abstract

**Background:**

There remains a lack of large-scale clinical studies of cognitive impairment that aim to increase diagnostic and prognostic accuracy as well as validate previous research findings. The MemClin project will amass large quantities of cross-disciplinary data allowing for the construction of robust models to improve diagnostic accuracy, expand our knowledge on differential diagnostics, strengthen longitudinal prognosis, and harmonise examination protocols across centres. The current article describes the Memory Clinic (MemClin) project’s study-design, materials and methods, and patient characteristics. In addition, we present preliminary descriptive data from the ongoing data collection.

**Methods:**

Nine out of ten memory clinics in the greater Stockholm area, which largely use the same examination methods, are included. The data collection of patients with different stages of cognitive impairment and dementia is coordinated centrally allowing for efficient and secure large-scale database construction. The MemClin project rest directly on the memory clinics examinations with cognitive measures, health parameters, and biomarkers.

**Results:**

Currently, the MemClin project has informed consent from 1543 patients. Herein, we present preliminary data from 835 patients with confirmed cognitive diagnosis and neuropsychological test data available. Of those, 239 had dementia, 487 mild cognitive impairment (MCI), and 104 subjective cognitive impairment (SCI). In addition, we present descriptive data on visual ratings of brain atrophy and cerebrospinal fluid markers.

**Conclusions:**

Based on our current progress and preliminary data, the MemClin project has a high potential to provide a large-scale database of 1200–1500 new patients annually. This coordinated data collection will allow for the construction of improved diagnostic and prognostic models for neurodegenerative disorders and other cognitive conditions in their naturalistic setting.

## Introduction

Here, we introduce a new clinical multi-centre study (Memory Clinic: MemClin) intended to fill the gap for large-scale clinical studies aimed at describing early stages of cognitive decline regarding diagnostic accuracy, longitudinal prognosis, differential diagnostics, and at creating better harmonised examination protocols.

The risk of dementia rises sharply with age [1], and according to the United Nations the aging population is increasing [2]. Dementia affects approximately 5% of the world’s elderly population [1], and the percentage of people with Alzheimer’s disease (AD) increases exponentially: 3% between 65 and 74 years, 17% between 75 and 84 years, and 32% older than 85 years [3]. These statistics describe the magnitude of the burden of cognitive disorders on the health-care system [3]. Decline in cognitive functions is also a general feature of normal aging reflected by deterioration in cognitive domains such as episodic memory [4], processing speed, and executive functions [5, 6]. A great clinical challenge is to accurately distinguish between normal age-related cognitive decline and the earliest signs of dementia. In keeping with that notion, the concept of mild cognitive impairment (MCI) was initially established as a prodrome for persons at elevated risk of developing AD [7]. MCI has been verified as a risk factor for dementia [8, 9], but nonetheless some MCI individuals remain stable or recover a normal cognitive status over time [10]. Recently, criteria for subjective cognitive decline (SCD) have been proposed [11, 12]. SCD is a concept contingent on self-reported cognitive decline but with unimpaired performance on cognitive test measures. In addition, SCD refers to progressive change from a previous cognitive level and not just an isolated complaint. It has been shown that SCD predicts dementia [13, 14]. However, MCI and SCD individuals may have a heterogeneous aetiology for cognitive impairment in early phases, with diverse clinical manifestations and different temporal trajectories of both their somatic and cognitive profiles. Thus, the cognitive and physiological trajectories in both SCD and MCI patients’ need to be explored further. In the current article, given the insufficient knowledge about change over time at the baseline examination, the term subjective cognitive impairment (SCI) will be used instead of SCD.

Misdiagnosis of dementia is an important clinical problem affecting the patient’s life and may have profound implications for their close relations. Increased knowledge on how neuropathological features relate to clinical criteria is urgently needed to prevent such outcomes [15]. To increase knowledge of the pathophysiology in dementias such as AD, biomarkers have been increasingly important [16, 17]. In keeping with that, an unbiased classification scheme (A/T/N) for AD has been proposed [18]. We have recently shown that the A/T/N scheme is a promising tool for increasing the degree of certainty in diagnostic and prognostic procedures when used in conjunction with information on brain atrophy (as assessed with magnetic resonance imaging (MRI)) [19]. However, the A/T/N scheme and other research findings need to be further validated in large-scale community cohorts such as MemClin. Due to potential heterogeneity of consensus procedures, and comorbidities often seen in memory clinics, these are perfect settings for investigating the validity of the A/T/N biomarker scheme regarding non-AD related dementias, injuries and somatic conditions. There are large-scale databases [20–23] on cognitive impairment, in research settings such as the Alzheimer’s disease neuroimaging initiative (ADNI) [24], in clinical settings such as the Gothenburg MCI study [25]. In comparison, the MemClin project strength is that it covers 9 out of 10 memory clinics in a city population greater than two million inhabitants. Thus, this is a naturalistic study with an unselected inclusion, and due to the magnitude of MemClin, it will be possible to regularly evaluate different aspects of the more unusual cognitive conditions, and neurodegenerative dementias such as dementia with Lewy Bodies, Parkinson’s disease dementia, subtypes of fronto-temporal dementias, and patients with dementia whose clinical diagnosis cannot be specified (“UNS”). This will contribute important information on these subtypes of dementia pathology. Also including these subtypes will provide a rare ability to generalise findings from MemClin to the general clinical population with cognitive impairment, for instance through improved differential diagnostics. Importantly, MemClin has the ability to follow the diagnostic trajectories of patients with SCI and MCI. Taken together, our ongoing MemClin project has a large-scale magnitude and population-based multi-centre character is envisioned to provide a platform concerning clinically relevant research for many years to come, targeting early clinical manifestations of diverse neurodegenerative disorders and other cognitive conditions. The MemClin research project will address three main objectives:

**(I)** To examine how cognitive profile, brain imaging [MRI and/or computed tomography (CT)], cerebrospinal fluid (CSF) biomarker candidates, and other health assessments (such as blood sampling and body-mass index) predict the longitudinal progression from SCI, MCI to dementia.

**(II)** To examine how specific cognitive tests, brain imaging, CSF, and other health assessments and the combination of those, relate to differential diagnostics in various cognitive stages and neurodegenerative disorders.

**(III)** To examine specific sub-types (e.g., interactions of cardiovascular pathology to AD pathology as well as atrophy patterns) of AD with respect to progression and diagnostic accuracy, but also to increase knowledge of “unspecified dementias (UNS)” (i.e., when a specific clinical diagnosis cannot be established).

The main objective of the current article is to describe the study design, materials and methods, and patient characterisation of the MemClin project. In addition, we will report descriptive data from the ongoing data collection.

## Methods

The different memory clinics in the greater Stockholm catchment area predominantly apply the same examination methods, which make centralized data-collection and merging of data possible. The MemClin project will build on clinical examinations by coordinating data collection between memory clinics, all contributing to one large-scale database. The project will receive data from 9 out of 10 independent memory clinics in Stockholm.

### Participating centres

**Fig. 1:**
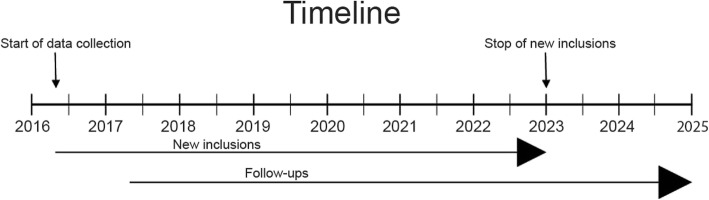
Preliminary timeline of the data collection

The prospective data collection is ongoing and began with the first memory clinic in April 2016 progressively followed by the other 8 clinics. The currently included memory clinics (Managing organization) are: Bromma memory clinic (Stockholm Sjukhem), Nacka memory clinic (Praktikertjänst), Dalen’s memory clinic (Praktikertjänst), Handen memory clinic (Praktikertjänst), Löwenströmska memory clinic (Capio), Sabbatsberg geriatric clinic (Stockholm County Council), Jakobsberg memory clinic (Stockholm County Council), and the memory clinics at Karolinska university hospital at Huddinge and Solna (both operated by Stockholm County Council). Patients are recruited from the Stockholm County and its suburb areas. A total of 2.102.788 out of 2.315.612 (information from statistics Sweden [SCB]) inhabitants constitute the MemClin catchment area. Inclusion of new cases will proceed through December 2021 (Fig. 1).

### Study design

**Fig. 2:**
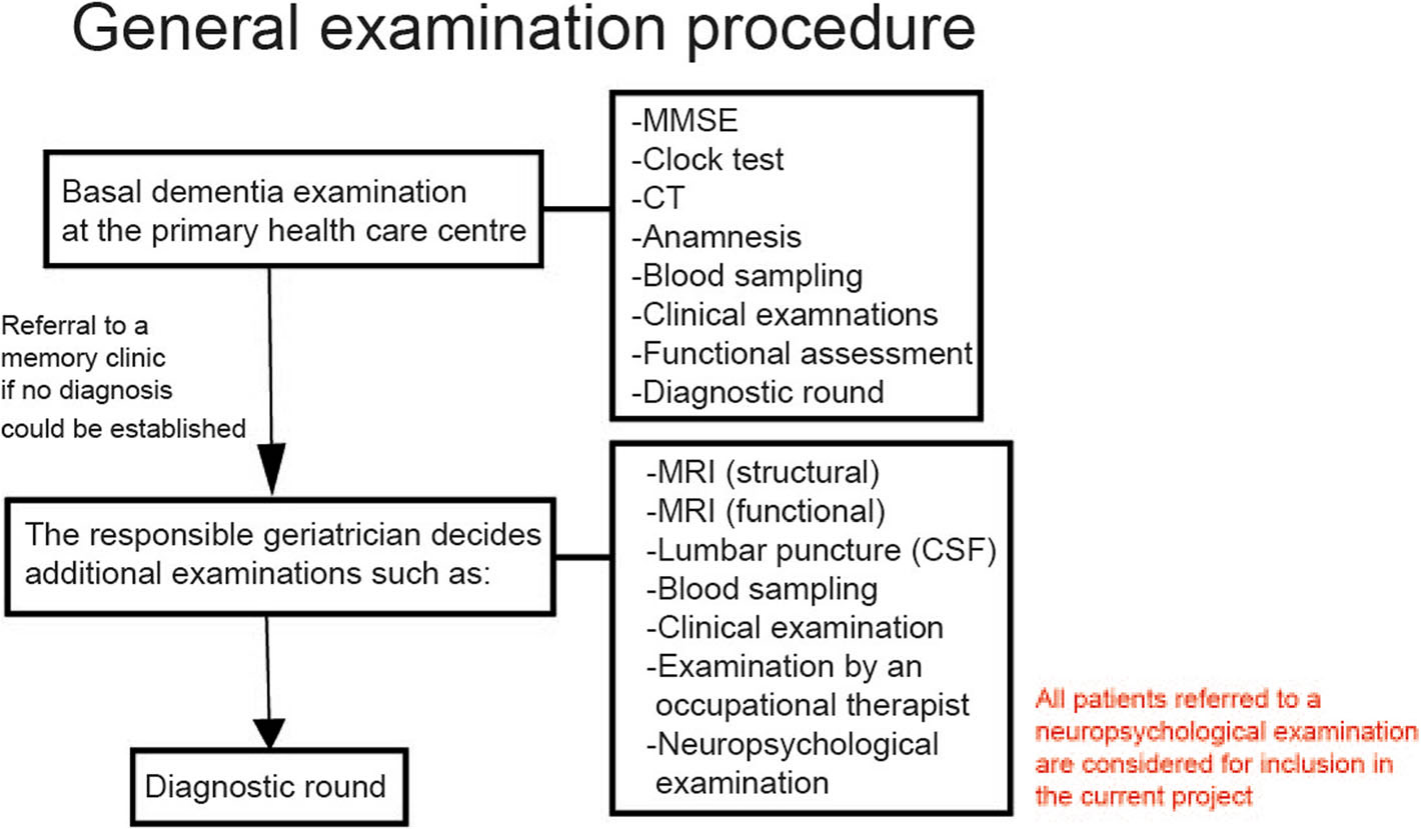
General examination procedure. MMSE = Mini Mental State Examination. CT = Computer Tomography. MRI = Magnetic Resonance Imaging. CSF = Cerebrospinal fluid. A few of the participants are not referred from the primary healthcare but from other specialist units such as the neurology department from regional care givers. In those cases, the participants might have to undergo additional examinations that relate to the basic dementia examination

Prior to the memory clinic examination and inclusion in the current research project, most patients have been assessed using a basic dementia examination in the primary healthcare. The examination is performed according to the guidelines established by the Swedish Board of Health and Welfare in 2010, revised in 2017 [26]. The basic examination generally includes a cognitive screening (Mini Mental State Examination [MMSE], and clock test), patient history, blood sampling, evaluation of health and functional status, and a head CT (a few patients are also examined with brain MRI) examination (Fig. 2). If the primary care facility is unable to determine a diagnosis, the patient is commonly referred to a memory clinic for extended examination. A small number of patients are also referred to the memory clinics from other healthcare specialist units such as Neurology clinics. At the memory clinic, the responsible geriatrician decides which additional examinations are needed to determine diagnosis (Fig. 2). All patients referred to neuropsychological examination at a memory clinic are considered for inclusion in the MemClin project. The examining psychologist may use clinical judgement to forgo asking the patient for participation. Only data from the memory clinic examination will be evaluated in the MemClin project (i.e., no additional research examinations will be conducted). Consequently, the extent of data collected may slightly vary between patients because the choice of evaluation methods for each specific patient is made by the psychologist and the responsible geriatrician. Thus, the MemClin project don’t specifically interfere in the clinical choices of examination methods. Nonetheless, the examination procedure is conducted in accordance with the Swedish National Board of Health and Welfare guidelines [26]. Diagnosis are determined at the multidisciplinary consensus meeting at the respective memory clinic. The clinical team at the memory clinic sets the cognitive diagnosis according to the ICD-10 [27], and in conjunction with the consensus diagnostic criteria for MCI [10]. Thus, all clinics apply the same diagnostic criteria. The diagnosis MCI is considered if the patient has evidence of cognitive decline, by self and/or informant report, and display deficits on objective cognitive measures during neuropsychological assessment. In addition, the patient should not fulfil diagnostic criteria for dementia and functional activities should be mainly preserved. The SCI patients are grouped according the ICD-10 diagnoses (no impairment on objective clinical measures): Z03.2 (observation for suspected mental and behavioural disorders), Z03.3 (observation for suspected nervous system disorders), Z03.8 (observation for other suspected diseases and conditions), and R41.8 (other and unspecified symptoms and signs involving cognitive functions and awareness).

### Participants

**Fig. 3:**
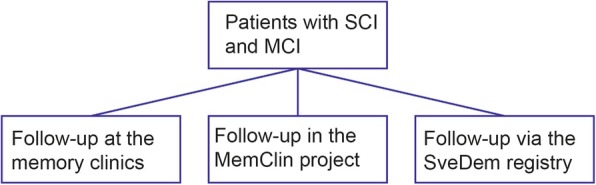
Flow-chart that illustrate the different paths of SCI follow-ups in order to decrease the amount of missing data

The MMSE score is not a criterion for inclusion since the patients referred to a neuropsychological examination are often in their early cognitive decline and thus tend to have MMSE scores above 24. All patients have subjective cognitive complaints either self-reported, or reported by relatives, friends, or members within their healthcare support system. Demographics and socioeconomic factors such as age, gender, and education (level and total years) are collected in addition to the clinical measures. Information about patient anamnesis, disease history, and neurological status will also be extracted from electronic health records. As of 2019, data on 1543 patients have been collected, and our approximation is that data for 1200–1500 new cases will be gathered annually. Some memory clinics regularly follow their patients across time, whereas other clinics complete their examination when a patient get a SCI or MCI an await a new referral from the primary health care (which may never occur). Due to this potential inconsistency between centers, we are planning several paths for follow-up (Fig. 3). 1. Approximately 250–400 cases will be re-examined at the memory clinics after 1–1 ½ year due to uncertain diagnostic status (i.e., SCI or some MCI cases) at the baseline examination. Of those 250–400 re-examined cases, approximately 50–75 cases will be re-examined at an additional occasion after 1–1 ½ year. 2. SCI patients that are not followed via the memory clinics will be contacted by the MemClin project and offered a full re-examination. This procedure will follow approximately 100 SCI patients annually. 3. Finally, those patients that the MemClin project will not be able to follow-up over time, may still become available for diagnostic follow-up via the SveDem registry [28] once (and if) they develop dementia.

### Assessments

#### Cognitive testing

Small individual variations can sometimes occur due to clinical organisation and the specific psychologist’s preferences. Once every semester, the neuropsychologists at the Memory Clinic network in Stockholm meet each other to harmonize examination methods and to discuss methodological issues. The MemClin project also invite the neuropsychologists once every semester to discuss the progress of the project and to share methodological issues. The aim is to reduce potential inconsistencies. In addition, the MemClin project will conduct control analysis to evaluate if inconsistencies between clinics interact with the results of interest. The project assesses a broad range of cognitive domains:

##### Episodic memory

Ray Auditory Verbal Learning Test (RAVLT) [29], Rey Complex Figure Test (RCFT) [30].

##### Executive functions

Trail Making Test (TMT) trial 4 from the Delis-Kaplan Executive Function System (D-KEFS), Verbal Fluency (VF) from the D-KEFS [31].

##### Attention/working-memory

Digit Span from the Wechsler Adult Intelligence Scale (WAIS)^4th^ [32], Trail Making Test (TMT) trial 1–3, and 5 from the D-KEFS [31].

##### Visuospatial/visuoconstructive functions

Block Design from the WAIS [32], Rey Complex Figure Test (RCFT).

##### Language

Boston Naming Test (BNT) [33].

##### Premorbid abilities/semantic memory

Information from the WAIS-IV [32].

In addition, clinicians at a majority of the memory clinics administer of the following tests: Logical Memory Test from the Wechsler Memory Scale-3rd, and Arithmetic, Matrix Reasoning, and Coding from the WAIS-IV, etc.

#### Brain imaging

Brain imaging data is collected from different brain imaging centres in Stockholm. The patients either get a high-resolution head CT or/and a brain MRI examination including a high resolution sagittal T1-weighted sequence for structural imaging (integrity of grey and white matter structures), and a T2-weighted sequence for additional contrast between CSF and tissue to exclude other medical causes, and a fluid-attenuated inversion recovery (FLAIR) sequence for visualising white matter lesions. Both 1.5 T, and 3 T scanners are used for MRI acquisitions. The collected imaging-data are analysed by a radiologist by using visual assessment scales such as the Scheltens scale for medial temporal atrophy (MTA; scored from zero to four) [34], the global cortical atrophy (GCA; scored from zero to three) scale [35], and the Fazekas scale for white matter changes (scored from zero to three) [36]. Higher scores in the scales denote end-stage of atrophy or greater white matter changes. The radiologists also analyse the images in order to detect potential ischemia or other structural abnormalities.

#### CSF biomarkers

An additional important aim of the research project is to analyse data from the CSF samples (β-amyloid, tau, p-tau), which is part of the diagnosis procedure. The lumbar puncture examinations are conducted by a physician affiliated to the specific memory clinic. The pathological cut-offs at the memory clinics are: ≤ 550 ng/l for β-amyloid, ≥ 80 ng/l for p-tau, and ≥ 400 ng/l for t-tau. No biological CSF data will be saved as a part of the research project. Only the numeric values referred from the examinations within the clinical routine will be handled, thus we will not conduct any additional CSF analysis.

#### Other measures

Other measures consist of blood samples (such as calcium, creatinine, C-reactive protein, APOE, homocysteine, blood sugar, and cholesterol-values etc.), blood pressure (systolic and diastolic), body-mass index (BMI), and disease history as well as information on comorbidity.

### Database system

All data collected throughout the course of the MemClin project will be stored in an instance of TheHiveDB database system [37]. TheHiveDB is an encrypted web-based neuroimaging database system capable of managing data for large longitudinal multi-centre projects. Data from different modalities (imaging, clinical, etc) will be aggregated by the system to provide a complete repository for data analysis. The system also performs fully automated analysis of imaging data using a variety of algorithms. These algorithms include standard processing pipelines and new machine learning based algorithms for visual ratings, which will help to overcome issues with inter-rater reliability and missing ratings from the participating radiologists.

### Statistical analysis

For the current manuscript, we solely present descriptive data and summary statistics for demographic, cognitive, and biomarker measures. The data was processed using SPSS.

## Results

At present, the MemClin project has obtained informed consent from 1543 patients. Only patients that have received their final diagnosis are included in the current report (*n* = 835). 708 patients are still under examination and awaiting their diagnosis.

In Table 1 we present demographic data and the number of patients by diagnostic group. For clarification, in the DLB/PDD group (dementia with Lewy-Bodies/Parkinson’s disease dementia), four had DLB and eight had PDD. In the group with fronto-temporal dementia, two had the behavioural variant, one had progressive non-fluent aphasia, one had Pick’s disease, and one had semantic dementia. In the group with other dementia, one had senile degeneration (ICD G311), and one had dementia not otherwise specified (ICD F028) together with sequelae of cerebral infarction (ICD I693). Lastly, we excluded five patients from the descriptive presentation because they had other conditions (not SCI, MCI, or dementia) that included conditions such as psychiatric, behavioural, alcohol related, other amnesia etc.

**Table 1:** Sample description

	AD	AD/Vasc.	Vasc.	DLB/PDD	FTD	UNS dem.	Other dem.	MCI	SCI
Number of patients	73	79	55	12	5	13	2	487	104
Age (years)	78,89 (6,34)	80,86 (6,01)	80,53 (6,02)	75,39 (3,99)	73,9 (4,72)	74,72 (5,65)	74,95 (3,32)	77,82 (6,02)	75,13 (5,66)
Education (years)	12,66 (3,52)	10,89 (3,52)	11,58 (2,99)	12,04 (3,06)	14 (3,39)	12,85 (3,74)	8,5 (2,12)	12,4 (3,62)	13,88 (3,94)
Cognitive screening MMSE	24,64 (2,47)	24,48 (2,99)	25,57 (2,26)	25,64 (3,53)	26,5 (0,58)	25,31 (2,66)	26 (1,41)	27,38 (2,06)	28,87 (1,48)
Gender (number of male/female)	28/45	42/37	29/26	8/4	2/3	9/4	2/0	255/232	41/63

Mean and SD. *AD* Alzheimer’s disease, *Vasc* Vascular dementia, *DLB* Lewy-Body dementia, *PDD* Parkinson’s disease dementia, *FTD* fronto-temporal dementia, *Dem* dementia, *UNS* unspecified, *MCI* mild cognitive impairment, *SCI* subjective cognitive impairment (no impairment on objective measures), *MMSE* mini mental state examination

### Cognitive performances

**Fig. 4:**
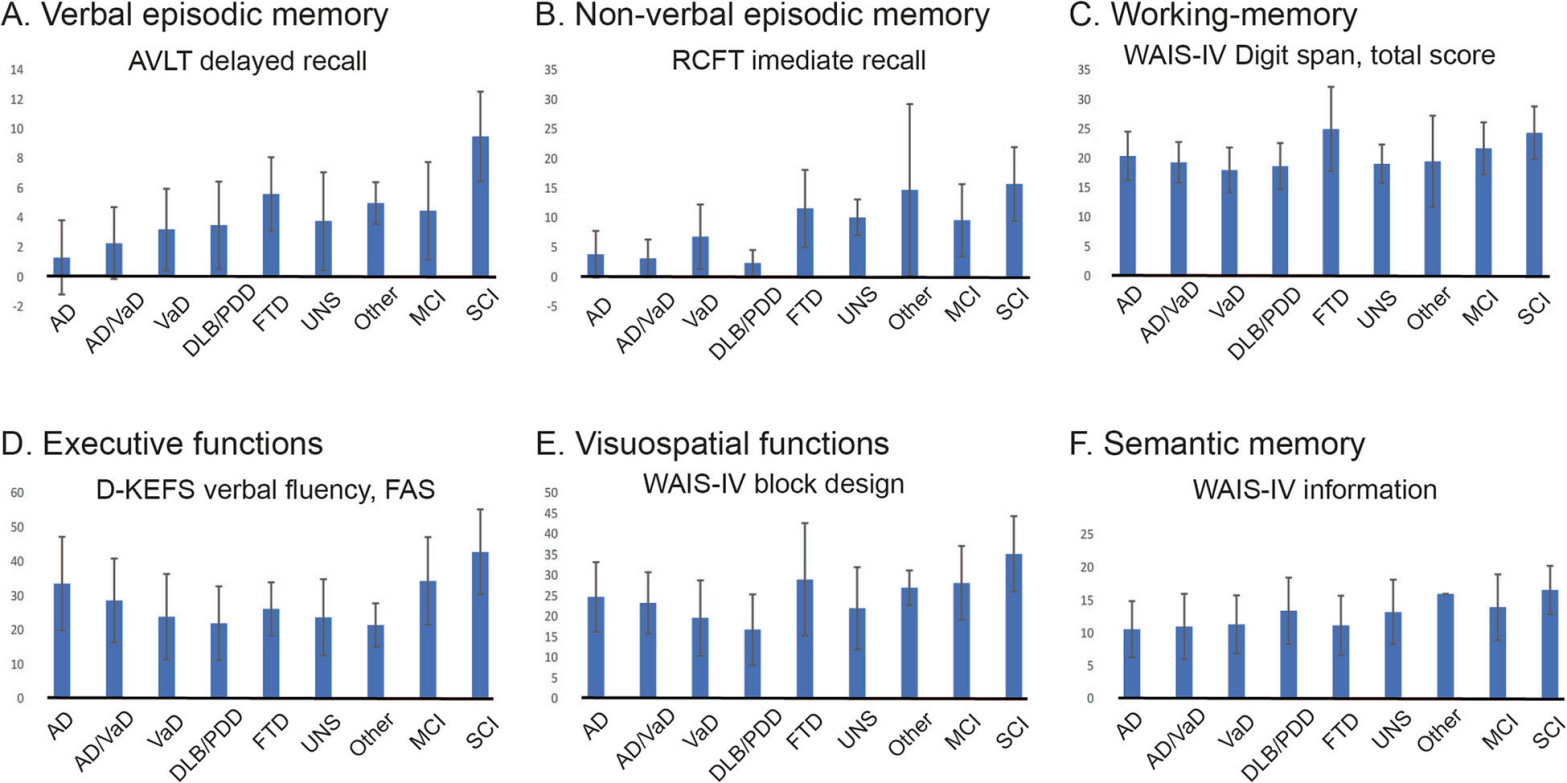
Group means of selected neuropsychological test measures **a**-**f** = Cognitive domains. Y-axis = Cognitive raw scores. X-axis = Cognitive diagnoses. AD = Alzheimer’s disease. VaD = Vascular dementia. DLB = Lewy-body dementia. PDD = Parkinson’s disease dementia. FTD = Fronto-temporal dementias. UNS = Unspecified dementia. Other = Other dementias. MCI = mild cognitive impairment. SCI = subjective cognitive impairment. AVLT = Auditory Verbal Learning Test. RCFT = Rey Complex Figure Test. WAIS = Wechsler Adult Intelligence Scale, 4^th^ edition. D-KEFS = Delis-Kaplan Executive Function System. FAS = letters F, A, S. Error bars = standard deviation

In Fig. 4, we present data within some of the cognitive domains. Generally, the SCI group performed better than the other groups, MCI performed intermediate, and both AD and vascular dementia performed the worst.

### Visual ratings of brain atrophy and CSF biomarkers

**Fig. 5:**
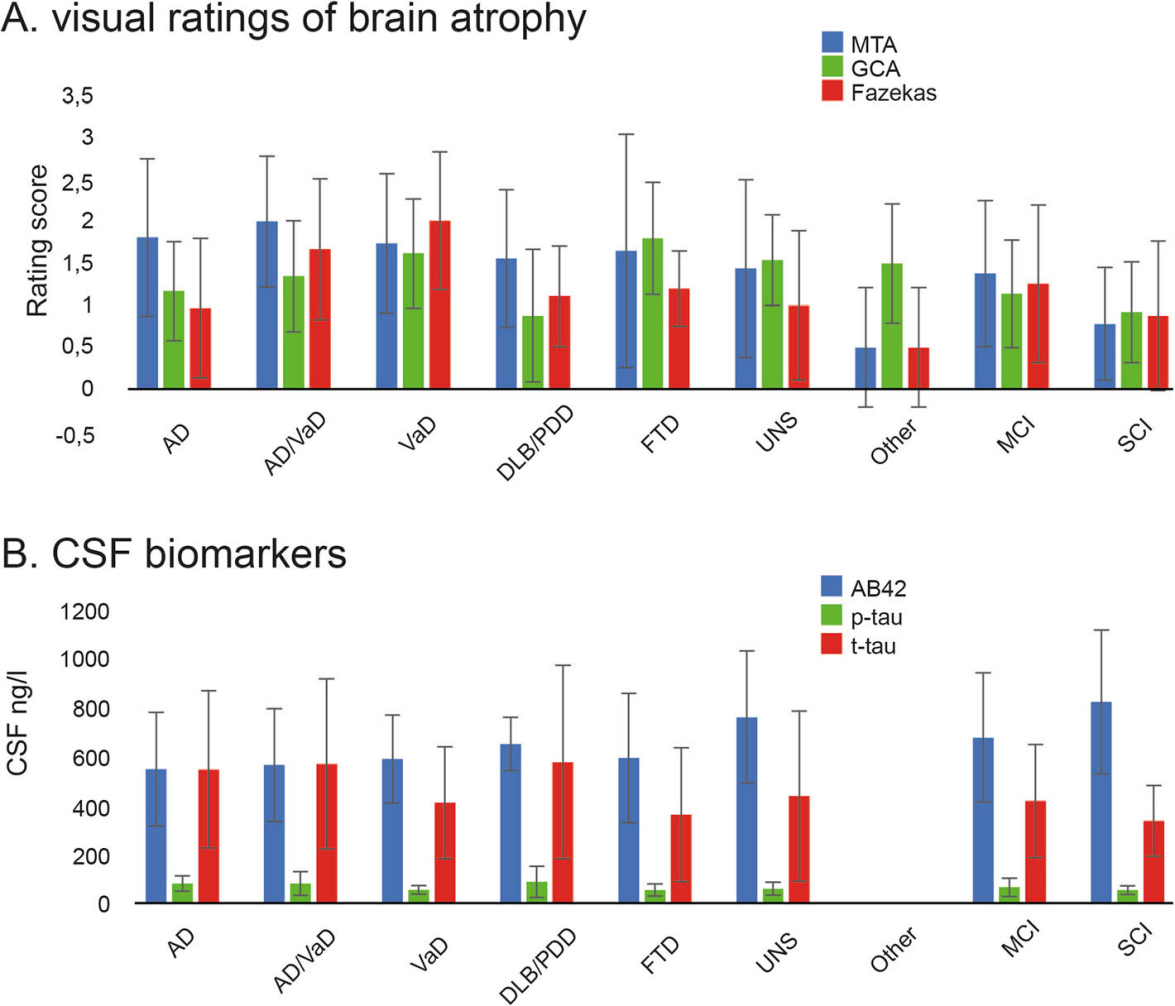
Visual ratings and cerebrospinal fluid biomarkers **a**, Visual ratings of brain atrophy. MTA = medial temporal atrophy. GCA = global cortical atrophy. **b**, Cerebrospinal fluid (CSF) biomarkers. Aβ = amyloid beta. p-tau = phosphorylated-tau. t-tau = total-tau. Ng/l = nanogram/liter. The abbreviations at the x-axis are referred to as: AD = Alzheimer’s disease. VaD = Vascular dementia. DLB= Lewy-body dementia. PDD = Parkinson’s disease dementia. FTD = Fronto-temporal dementias. UNS = Unspecified dementia. Other = Other dementias. MCI = mild cognitive impairment. SCI = subjective cognitive impairment. Error bars = standard deviation

In Fig. 5, we present measures of visual ratings derived from CT/MRI, as well as for the CSF biomarkers. For MTA, 516 patients (62%) had a CT-scan and 155 (19%) had an MRI-scan. For GCA, 515 (62%) had a CT-scan and 153 (18%) had an MRI-scan. Finally, for Fazekas, 508 (61%) had a CT-scan and 152 (18%) had an MRI-scan. For the CSF biomarkers, 357 patients had Aβ42 data, 356 had p-tau, and 355 had t-tau (43% on all CSF biomarkers). Generally, the SCI group had the lowest ratings (i.e., less brain atrophy) of brain atrophy, the MCI group had intermediate ratings, and the groups with AD and vascular dementia had the highest ratings (i.e., more atrophy). In addition, the SCI group had the highest Aβ42 values, the MCI group had intermediate values, and the groups with AD and vascular dementia had the lowest Aβ42 values.

## Discussion

The main purpose of this article was to introduce the ongoing MemClin project in which we coordinate data collection across nine out of ten memory clinics in the greater Stockholm catchment area in Sweden. We also briefly presented patient characteristics and preliminary descriptive results. At present, the data collection of the MemClin project is in progress and more than 1500 patients are currently included. We expect to be collecting data in full scale throughout 2019–2022, gathering data for approximately 1200–1500 new patients annually. All memory clinics in the greater Stockholm area largely use similar examination methods, which facilitates data collection and aggregation. Establishing a large-scale clinical database of relevant data will enable us to build the robust models required to improve diagnostic dementia accuracy, differential diagnostics, and hopefully shorten the time from initial exam to a reliable diagnosis. Ultimately, the MemClin effort serves the overarching purpose of better and more cost-efficient healthcare for future memory clinics.

The preliminary data herein was in line with the expected outcome. This was for example illustrated as overall superior cognitive performances observed in SCI patients, intermediate cognitive performances in MCI patients, and inferior performances on cognitive measures such as episodic memory scores in patients with AD. Additional analyses will be conducted when the MemClin project can provide increased statistical power and more advanced statistical modelling.

There are large-scale databases available that aim to improve quality of dementia care by evaluating adherence to national guidelines and support care harmonisation across clinics, such as the SveDem national quality registry [28]. However, those registries do not provide information on extensive neuropsychological measures and early phase cognitive impairment (i.e., SCI and MCI). MemClin will provide this essential information. Furthermore, due to the magnitude of MemClin, it will be possible to regularly evaluate different aspects of the more unusual neurodegenerative dementias such as dementia with Lewy Bodies, Parkinson’s disease dementia, subtypes of fronto-temporal dementias, and patients with dementia whose clinical diagnosis cannot be specified (“UNS”). This will contribute important information n these subtypes of dementia pathology. Also including these subtypes will provide a rare ability to generalise findings from MemClin to the general clinical population with cognitive impairment, for instance through improved differential diagnostics. Most importantly, the project will generate harmonised examination protocols with highest possible sensitivity to prodromal stages of dementias. By harmonising diagnostic procedures across clinics, the MemClin effort serves the overarching purpose of better and more cost-efficient healthcare for future memory clinics. Furthermore, the knowledge generated from MemClin will provide dementia examinations with a higher quality and diagnostic accuracy and thus contribute to a more adequate caretaking and less acute healthcare arrangements. This improvement will have implications on the quality and accuracy of dementia diagnosis within the Stockholm catchment area, and also potentially domestically and internationally. Lastly, because data collection is conducted as part of the clinical routine, new reliable findings can be applied back to the clinics within a relatively short timeframe of implementation.

There are some limitations to the current study. Clinicians at these memory clinics use visual ratings (CT/MRI) from clinical radiologists and thus we introduce unknown variability that could affect inter-rater reliability. Further, at present we are missing values for the visual rating scales of some patients despite having CT/MRI data available. Therefore, we will re-evaluate the ratings with expert neuroradiologists to harmonise the ratings, minimise inter-rater disagreement, and provide comparable atrophy estimates for all MemClin CT/MRI data. We also plan to conduct automated brain-imaging analyses on these images [38]. Since this is a multi-centre study there will be different practices and priorities at the centres when it comes to cognitive testing, CSF sampling, additional CT/MRI evaluations, and other health measures in relation to the follows. Due to this potential inconsistency between centres, we are planning to follow the patients with SCI within the MemClin project to receive comparable and useful data for those in the earliest phases of cognitive impairments. In addition, those patients that the MemClin project will not be able to follow over time, may still become available for diagnostic follow via the SveDem registry once they develop dementia [28]. We are aware that potential biases can occur considering incomplete data that effect the validity of the research results. Our approach to follow the diagnostic trajectories in patients with SCI from different sources will decrease the risk for biases. However, we will also conduct sensitivity analyses to assess the effect of dropout and missing data.

Since part of the reported data in the MemClin project are included in the diagnostic criteria, there is a risk of circularity bias. Therefore, circularity bias needs to be considered when examining the diagnostic estimates. Cognitive tests are included in the diagnostic criteria. CSF biomarkers are used for the diagnosis in approximately 40% of the MemClin patients. MRI and/or CT are used in an unstructured way to ascertain brain atrophy and exclude tumours, etc. in approximately 80% of the MemClin patients. Thus, there may be certain risk of circularity bias when addressing our project’s aims I and II, especially for cognitive tests and visual ratings from MRI/CT. Therefore, we will consider this circularity bias when examining the diagnostic estimates, for example by comparing the value of measures at different levels of bias (e.g. cognitive tests vs. CSF) as well as by re-diagnosing patients for specific studies.

## Conclusions

We have here introduced the MemClin project in respect of study design materials, methods, and patient characterisation. In addition, we have reported descriptive data from the ongoing data collection. The naturalistic MemClin project will provide a variety of assessment data from the memory clinic examinations in the Stockholm catchment area. The coordinated data collection will help us establish a large-scale database that can be used to build robust models to improve diagnostic accuracy of dementia and other cognitive conditions. Importantly, in the long run the MemClin project will create harmonised examination protocols with highest possible sensitivity to track and differentiate dementias in early stages.

## Data Availability

The data which support this study are not publicly available. However, the data may be available from the corresponding author upon reasonable request.

## Abbreviations

AD: Alzheimer’s disease
Aβ: Amyloid beta
CSF: Cerebrospinal fluid
CT: Computer tomography
D-KEFS: Delis-Kaplan Executive Function System
DLB: Lewy-Body dementia
GCA: Global Cortical Atrophy
MCI: Mild cognitive impairment
MMSE: Mini mental state examination
MRI: Magnetic resonance imaging
MTA: Medial Temporal Atrophy
PDD: Parkinson’s disease dementia
p-tau: Phosphorylated-tau
RAVLT: Rey auditory verbal learning test
RCFT: Rey complex figure test
SCD: Subjective cognitive decline
SCI: Subjective cognitive impairment
TMT: Trail Making Test
t-tau: Total-tau
VF: Verbal Fluency
WAIS: Wechsler Adult Intelligence Scale
